# 14 Years after Discovery: Clinical Follow-up on 15 Patients with Inducible Co-Stimulator Deficiency

**DOI:** 10.3389/fimmu.2017.00964

**Published:** 2017-08-16

**Authors:** Johanna Schepp, Janet Chou, Andrea Skrabl-Baumgartner, Peter D. Arkwright, Karin R. Engelhardt, Sophie Hambleton, Tomohiro Morio, Ekkehard Röther, Klaus Warnatz, Raif Geha, Bodo Grimbacher

**Affiliations:** ^1^Center for Chronic Immunodeficiency (CCI), Medical Center, Faculty of Medicine, University of Freiburg, Freiburg, Germany; ^2^Division of Immunology, Boston Children’s Hospital, Department of Pediatrics, Harvard Medical School, Boston, MA, United States; ^3^Department of Paediatrics and Adolescent Medicine, Medical University of Graz, Graz, Austria; ^4^Royal Manchester Children’s Hospital, University of Manchester, Manchester, United Kingdom; ^5^Primary Immunodeficiency Group, Institute of Cellular Medicine, Newcastle University, Newcastle upon Tyne, United Kingdom; ^6^Great North Children’s Hospital, Newcastle upon Tyne Hospitals, NHS Foundation Trust, Newcastle upon Tyne, United Kingdom; ^7^Department of Pediatrics and Developmental Biology, Graduate School of Medical and Dental Sciences, Tokyo Medical and Dental University, Tokyo, Japan; ^8^Rheumatology Medical Center, Villingen-Schwenningen, Germany; ^9^Institute of Immunology and Transplantation, Royal Free Hospital, University College London, London, United Kingdom

**Keywords:** ICOS deficiency, common variable immunodeficiency, hypogammaglobulinemia, autoimmunity, immune dysregulation, combined immunodeficiency, opportunistic infections

## Abstract

**Background:**

Inducible co-stimulator (ICOS) deficiency was the first monogenic defect reported to cause common variable immunodeficiency (CVID)-like disease in 2003. Since then, 16 patients have been reported worldwide with an increasing range of clinical phenotypes.

**Objective:**

We sought to compare the clinical and immunological phenotype and provide clinical follow-up and therapeutic approaches for treating ICOS-deficient patients.

**Methods:**

We describe the clinical and laboratory data of 15 patients with available clinical data. Previous publications and clinical assessment were used as data sources.

**Results:**

The observed ICOS gene mutations were all deletions leading to undetectable protein expression. The clinical phenotype of ICOS deficiency is much broader than initially anticipated and includes not only CVID-like disease but an increased susceptibility to viral and opportunistic infections, as well as cancer. Impaired B-cell development led to decreased memory B-cells in all patients, and hypogammaglobulinemia in all but one patient. Circulating CXCR5^+^ CD4^+^ follicular T-helper-cell numbers were also reduced in all patients. Treatment included immunoglobulin replacement, regular antibiotic prophylaxis, corticosteroids, and steroid-sparing agents. Three patients underwent hematopoietic stem cell transplantation; one of them died due to capillary leak syndrome on day 5 posttransplantation.

**Conclusion:**

The disease spectrum of ICOS deficiency is expanding from solely B-cell to combined B- and T-cell immunodeficiency, suggesting genetic and environmental modifiers. Genetic diagnosis is the only tool to distinguish ICOS deficiency from other immunological defects. Patients with antibody deficiency, autoimmunity, and combined immunodeficiency should be screened for *ICOS* mutations.

## Introduction

Common variable immunodeficiency (CVID) is the most prevalent primary immunodeficiency in adults (1) with an estimated incidence of 1:25,000–1:50,000 (2). Clinically, patients present with an increased susceptibility to infections, autoimmune manifestations, and an increased risk of malignancy (3). The disease is defined by a marked decrease (at least 2 SD below the mean for age) in serum immunoglobulin G (IgG) and IgA and/or IgM, and an age of greater than 4 years at diagnosis independent of onset (https://esid.org/Working-Parties/Registry/Diagnosis-criteria).

Over the last 14 years, various monogenic defects have been identified to be the cause of CVID-like disease (4), allowing for more precise diagnosis and comparability within the subgroups of this heterogeneous disease. Understanding the molecular mechanisms underlying CVID should lead to more specific treatment. The first described monogenic defect causing CVID-like disease was the lack of the inducible co-stimulator (ICOS). Following the initial description of this monogenic defect in 2003 (5), a number of other patients have been identified worldwide. Of the seven families, three came from Germany (5, 6), one from Austria (6), one from Japan (7), one from Kuwait (8), and one from UK with Pakistani background (9).

The *ICOS* gene has five exons with 2,620 nucleotides, encoding a 199-amino acid protein that forms a 55–60 kDa disulfide-linked homodimer. *ICOS* is located very close to the *CTLA-4* and *CD28* genes on chromosome 2q33–34 (10). It is exclusively upregulated on activated T-cells.

Like CD28, but unlike CTLA4 and programmed cell death protein 1, ICOS delivers a positive co-stimulatory signal to the T-cell (11). The functions of CD28 and ICOS are only partially overlapping. CD28 is constitutively expressed on naïve and mature T-cells, while ICOS expression is low on naïve T-cells and upregulated upon T-cell receptor stimulation (11). In spite of some differences in intracellular-associated molecules, CD28 and ICOS share common signaling pathways like phosphorylation of Erk1/2, protein kinase B, phosphoinositide-dependent kinase-1, p38 (12), and recruitment of phosphatidylinositol-3-kinase (PI3K) (13). Signaling through both ICOS and CD28 induces proliferation, cell survival, and differentiation. ICOS co-induces the secretion of IL-4, IL-5, IL-6, granulocyte-macrophage colony-stimulating factor, TNF-α, and interferon gamma (IFN-γ). Moreover, ICOS induces IL-10 secretion (14), whereas CD28 induces IL-2 (10). ICOS-L is the unique ligand of ICOS. ICOS-L is constitutively expressed on both lymphoid and non-lymphoid tissues. Inflammatory stimuli, such as TNF-α, IFN-γ, and lipopolysaccharide, lead to its upregulation (15). CD28, however, binds to CD80 and CD86, which are expressed by antigen presenting cells like B-cells, monocytes, macrophages, and immature dendritic cells (16). Finally, both CD28 and ICOS are crucial for the development and maintenance of regulatory T-cells (Tregs) (17, 18).

A distinct role of ICOS is the generation and maintenance of germinal centers (GCs) in lymphatic tissues. ICOS deficiency, therefore, results in deficient formation of memory B-cells and impaired switched antibody responses (19). ICOS is also considered to be a crucial survival signal for C–C chemokine receptor type 5 (CXCR5)-positive follicular T-helper-cells (TFH) (20). ICOS is highly expressed on those cells, which are primarily localized in the light zone of the GC (21). ICOS signaling is crucial for the migration of TFH-cells from the T–B cell border into the follicle. This is facilitated by two mechanisms: ICOS-dependent upregulation of the chemokine receptor CXCR5 (22) results in attraction of the T-cells toward the B-cell follicle *via* the chemokine ligand chemokine (C–X–C motif) ligand 13, which is secreted by follicular dendritic cells. Moreover, ICOS triggering has been shown to promote persistent motility of T-cells in a PI3K-dependent manner, independent of its co-stimulatory function (23). Costimulation *via* ICOS is thought to be responsible for the induction of IL-10 (24) and IL-21 in TFH (25). These cytokines are important for the maintenance of GCs and thereby the differentiation of B-cells into long-lived switched memory cells and plasma cells (26). This explains why ICOS deficiency classically manifests with a severely impaired memory B-cell compartment.

The phenotype of ICOS-deficient patients highlights various effects of this deficiency on their immune system. This paper provides an update on all 15 published patients diagnosed with ICOS deficiency to date and available clinical data. We sought to compare their clinical and immunological phenotype, and provide clinical follow-up and therapeutic approaches for treating ICOS-deficient patients.

## Materials and Methods

We describe the clinical and laboratory data of 15 patients with ICOS deficiency. Previous case reports as well as direct clinical evaluation of the patients by their attending physicians were used as source material. Data from one child of family 6, the twin sister of patient #12, was not reviewed, as she had died of a septic shock in infancy and little is known about her clinical condition. Written consent was obtained of all patients or their parents, respectively. This study was conducted under the approved ethics protocol No. 295/13, dated July 17, 2013, from the University clinic, Albert Ludwigs University Freiburg.

## Results

### Clinical Manifestations

**Figure 1:**
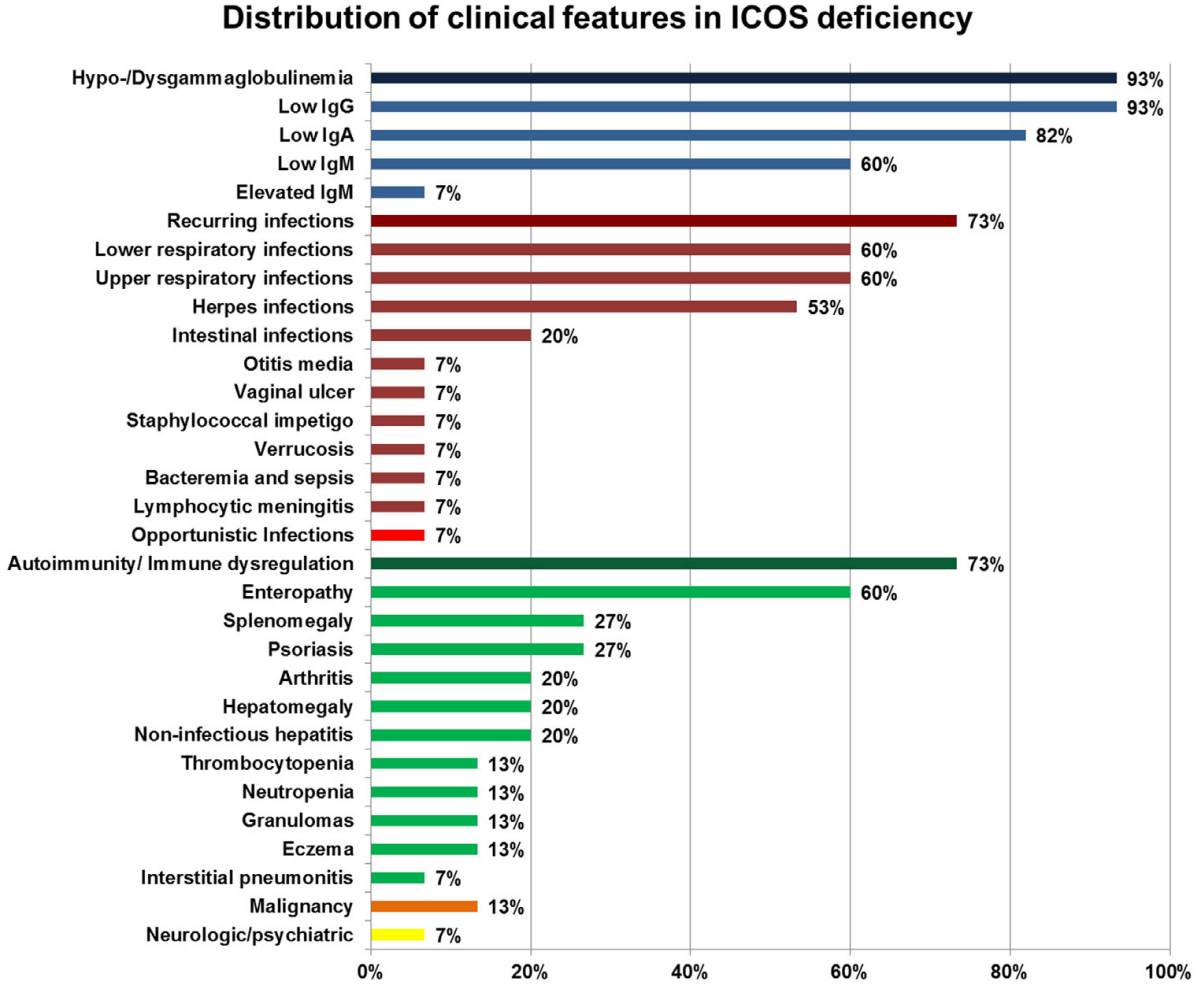
Distribution of clinical features in inducible co-stimulator (ICOS) deficiency. Percentages of a specific symptom depict the proportion of symptomatic patients within 15 ICOS-deficient patients. Deep blue bars represent hypo-/dysgammaglobulinemia, deep red bars cluster recurring infections, the bright red bar highlights opportunistic infections, green bars indicate autoimmunity and immune dysregulation, the orange bar represents malignancies, and the yellow bar indicates neurologic/psychiatric features. Immune dysregulation, autoimmune manifestations, inflammatory bowel disease, severe eczema, lymphoproliferation, granuloma (according to ESID Registry—Working Definitions for Clinical Diagnosis of PID).

Clinical features of ICOS deficiency varied significantly, including one asymptomatic patient (now aged 15). The average age of onset of symptoms was 14 years, ranging from 1 month to 35 years, with six patients only showing first symptoms in adulthood. One patient (#1) died at age 44 of malignancy [human papilloma virus (HPV)-associated vulvar carcinoma], one patient (#4) died at age 37 due to complications of an unrelated condition, and one patient (#14) died at age 6 years following a capillary leak syndrome on day 5 post hematopoietic stem cell transplantation (HSCT). The patients’ age at evaluation ranged from 3 to 56 years, with a total follow-up time of 122 years (ranging from 1 to 13 years). Six patients were female, 10 were male. An overview of the distribution of the clinical features is given in Figure 1. Supplementary text and Table S1 in Supplementary Material provide a detailed summary of the individual clinical manifestations and Table S2 in Supplementary Material laboratory values.

The infectious phenotype of the ICOS-deficient patients varied, but mainly consisted of bacterial and *Herpes simplex* or cytomegalovirus (CMV) infections. However, two patients also suffered from defective handling of a human herpesvirus 6 (HHV6) infection or an opportunistic *Pneumocystis jirovecii* infection, respectively.

Seventy-three percent (11/15) had recurrent infections. Respiratory infections were the predominant problem with 60% (9/15) of patients suffering from upper respiratory infections and lower respiratory infections including pneumonia. Lobectomy was performed in one patient (#7) following necrotizing pneumonia, and three patients suffered from chronic parenchymal lung damage such as bronchiectasis (#3, #7) and emphysema (#5).

Eight patients had recurrent Herpes virus infections including four cases of Herpes simplex (patient #2, #5, #6, and #7), four cases of CMV infection (patient #7, #9, #10, and #12), and one case of HHV6 (#14). The severity of these viral infections varied: two patients (#5 and #6) had moderate to severe recurring herpes labialis that could be controlled by topical treatment. Patient #2 had recurring herpes keratitis and developed an ulcer leading to keratoplasty at age 39 years, requiring treatment with oral prednisolone and valacyclovir. Patient #7 suffered of genital herpes at age 24 with strong local symptoms but no signs of systemic infection as in fever or elevated CRP levels. She was successfully treated with intravenous acyclovir. A mild relapse 1 year later was treated with oral valacyclovir. Patient #7 was diagnosed with CMV at age 28 in the context of an aggravation of her inflammatory bowel disease (IBD)-like symptoms. After oral valganciclovir treatment, CMV-specific PCR turned negative. Several reactivations detected by PCR positivity in blood, urine, and stools did not correlate with an exacerbation of symptoms and were consequently not treated. Her brother’s (patient #9) CMV infection first manifested with severe bloody diarrhea, fever, and hepatopathy at age 14. He required 7 days of intravenous aciclovir followed by 14 days of oral valganciclovir. Two reactivations were treated successfully with 14 and 21 days of valganciclovir. Patient #10 had recurrent CMV vulvovaginitis at age 45 requiring long-term oral valganciclovir. At age 48, she developed abdominal pain associated with bloody stools, and was diagnosed with CMV colitis by biopsy and by PCR on blood and the biopsy specimen. After 3 weeks of ganciclovir, all symptoms resolved. During an episode of *Candida*-induced acute respiratory failure and severe chronic diarrhea at age 1 month, patient #12 was also noted to have low-level CMV viremia. Posttransplant, he developed a CMV reactivation treated successfully with ganciclovir and CMV-specific immunoglobulin (Cytogam). One patient (#14) showed defective handling of HHV6 colitis. The virus was confirmed in samples from the sigmoid, duodenum, and liver. Following treatment with intravenous ganciclovir and then oral valganciclovir together with nitazoxanide, liver, and duodenal samples became negative for HHV6. However, a sigmoid biopsy remained positive and clinically, severe colitis associated with diarrhea and abdominal pain persisted.

Three patients suffered from other intestinal infections: *Campylobacter* enteritis in #2, *Salmonella* enteritis in #4, and norovirus, adenovirus, and *Cryptosporidium* enteritis in #14. Other infections included otitis media (#3), *Staphylococcus* impetigo (#1), severe verrucosis (#6), vaginal ulcer (#10), *Helicobacter cinaedi* bacteremia (#10), *Escherichia coli* sepsis (#10), and lymphocytic meningoencephalitis of unclear cause (#5). An opportunistic infection was observed in one patient (#12), who suffered from *Pneumocystis jirovecii* pneumonia at age two.

Autoimmunity and immune dysregulation were observed in 73% (11/15) of patients. Nine patients were affected by enteropathy, three by arthritis and one patient by interstitial pneumonitis (#10). Four patients developed splenomegaly (one accompanied by mild thrombocytopenia), and three hepatomegaly. Non-infectious hepatitis occurred in three patients: patient #1 had an alcoholic steatohepatitis. In patient #4, the pathogenesis of hepatitis remained unclear. Drug-induced toxicity was suspected according to histological findings and intake of a quinolone antibiotic shortly before. No serologic evidence of autoimmune hepatitis was found, and sarcoidosis was excluded histologically. In patient #15, a flare of hepatitis resolved spontaneously and a specific cause was not found either. Two each had thrombocytopenia and neutropenia, respectively. Four patients suffered from psoriasis and two patients had eczema. Two patients had granulomas: in one patient with chronic eczema, a biopsy showed a granulomatous inflammatory process with sarcoid-like lesions. In patient #1, multiple small non-caseating granulomas were found in one of two lymph nodes examined following lymphadenectomy after recurrence of her vulval carcinoma.

Two patients developed cancer: patient #1 developed a verrucous squamous epidermal carcinoma of the vulva associated with human papilloma virus infection at age 34 from which she died aged 44. In patient #6, large granular lymphocyte T-cells with clonal expansion were confirmed at age 51. In the same patient, a pre-auricular squamous cell carcinoma grade I was excised at age 53.

### Impaired Immune Function in ICOS-Deficient Patients

Inducible co-stimulator deficiency can affect the T-cell and the B-cell compartments. All patients had low TFH and low switched memory B-cells. Other abnormalities were not present in all patients, indicating phenotypic variability (see Table S2 in Supplementary Material).

#### T-Cell Phenotype

Although all patients presented with normal absolute peripheral T-cell counts, and immunophenotypic analysis was not uniform, the following T-cell subset abnormalities were observed: In families 1–4, 6, and 7, there was a normal distribution of naïve, memory, and effector T-cells, except for patients #2, #6, and #7 who had inverted CD4^+^/CD8^+^ ratios (19), and patient #12 from family 6 who had high CD8^+^ effector memory T-cells (TEMs). In family 5, however, there was a severe reduction in memory T-cell numbers. In these patients, further analysis of the memory subsets showed that CCR7^+^ CD62L^+^ CD45RO^+^ central memory T-cells as well as CCR7^−^CD62L^−^CD45RO^+^ TEMs were significantly reduced in both CD4^+^ as well as CD8^+^ T-cells (7).

In all families, a severe reduction of circulating TFH, defined as CXCR5^+^CD45RO^+^ memory CD4^+^ T-cells, was found, despite normal memory T-cell counts. This finding is in concordance with the fact that ICOS deficiency is associated with an impaired upregulation of CXCR5 on T-cells after stimulation (8, 9, 20).

FoxP3^+^ regulatory T cell counts were normal in the patients from families 1-4, family 5, and family 6 (7, 8). In contrast to the others, in family 5, there was a reduction of regulatory T cell FOXP3 expression as measured by mean fluorescence intensity, and a decrease in the CD45RO^+^CTLA4^+^ subset producing IL10, although, transforming growth factor beta-producing CD45RA^+^CTLA4dull^+^ cells were unaffected. The intensity of CTLA4 expression on the CTLA4^+^ subset was also diminished in those patients (7). These markers were not determined in the other families.

Patient #6 from family 3 developed an expansion of T-cell large granular lymphocyte cells with clonal restriction suspicious of lymphoma. He recently presented with a distinct expansion of double negative T-cells and CD8^+^ effector T-cells with elevated activation markers (HLA-DR), alongside a severe decrease of CD4^+^ T-cells, CD8^+^ T-cells, NK-cells, and ongoing B-cell lymphopenia. Within the CD8^+^ T-cell compartment, the naïve T-cells were severely decreased. For details on the patient’s clinical course, please see his case report in the Supplementary material.

#### B-Cell Phenotype

B-cell counts were decreased in all adult patients, whereas the children from family 4, 6, and 7 presented with normal B-cell counts upon diagnosis (8, 9, 19). However, when reanalyzed 12 years later after diagnosis, patients from family 4 also showed decreased B-cell counts. Two stages of B-cell development were disturbed in ICOS-deficient patients. In five of six adult patients in families 1–3, a severe reduction in absolute numbers of circulating naïve CD27^−^ B-cells was observed (19). Consecutive bone marrow biopsies of patients #3, #4 (19), and later on also in patient #5 showed a relative expansion of the cytoplasmic Ig-negative pre-B-I cells indicating a partial block of differentiation into the cytoplasmic Ig-positive pre-B-II cell stage. In contrast to previous reports (19), absolute numbers of naïve B-cells were now also decreased in the patients of family 4, and in patient #5, who now presented with low absolute B-cell counts as well as decreased naïve B-cell counts. They were also low in patient #10 from family 5, whereas her brother (patient #11) showed almost normal numbers of naïve B-cells at age 44, in spite of low total B-cell counts. In families 6 and 7, the children’s (aged from 3 to 11 years) normal total B-cell counts went along with high naïve B-cell numbers. Further B-cell development in ICOS-deficient patients seems unimpaired until the differentiation into class switched memory B-cells: CD19^+^CD27^+^IgD^−^ switched memory B-cells were markedly decreased at varying levels (but still detectable in 14/15 patients) in all ICOS-deficient patients (7–9, 19). Somatic hypermutation in the V region of the IgM gene was normal in three patients (19).

#### Immunoglobulins and Cytokines

In keeping with low class switched B-cells, hypogammaglobulinemia was observed in all but one patient (#13). Immunoglobulin levels are indicated in Table 1. IgG levels were consistently low in all patients but #13, who had low IgG levels at age 3 years but low normal values when re-evaluated at age 10. In ten patients, IgG levels were <3 g/L at diagnosis. 82% (12/15) had also low IgA levels, whereas IgM levels varied. 60% (9/15) had decreased IgM levels, five patients had normal, and one patient (#11) had elevated IgM levels. Specific antibody responses to vaccinations were not detectable, except for low levels of a phage-specific IgM in patient #1, a low titer of anti-diphtheria antibodies in patient #8, protective tetanus titers in patient #13, and measles and mumps IgG in patient #15. (Vaccination levels of patient #13 were not measured before immunoglobulin substitution was started). Anti-*Borrelia burgdorferi* IgM were detected once in the cerebrospinal fluid of patient #5.

**Table 1:** Immunoglobulin levels in [g/L] of inducible co-stimulator -deficient patients at the time of diagnosis [immunoglobulin G (IgG)] and after initiation of immunoglobulin substitution therapy (IgG, IgA, IgM).

Patients (age at measurement in years)	IgG on intravenous immunoglobulin (IVIG)/subcutaneous immunoglobulin	IgG without IVIG	IgA	IgM
Fam1—01 (44)	5.2	1.9	<0.25	<0.17
Fam1—02 (46)	6.2	2.9	0.27	<0.16
Fam2—03 (47)	2.4	1.1	0.14	0.1
Fam2—04 (37)	2.3	<1.4	0.05	0.55
Fam3—05 (56)	8.51	3.2	<0.06	0.29
Fam3—06 (52)	7.21	N/A	<0.07	0.97
Fam4—07 (28)	13.6	2.85	<0.06	<0.17
Fam4—08 (15)	3.54	1.54	0.6	0.27
Fam4—09 (15)	3.88	0.48	0.11	<0.17
Fam5—10 (47)	7.97	3.15	0.14	0.61
Fam5—11 (44)	6.5	6.11	0.1	*4.32*
Fam6—12 (2)	1.5	1.1	<0.007	0.84
Fam6—13 (10)	N/A^a^	6.5	N/A	N/A
Fam7—14 (5)	6.32	2.07	0.43	0.17
Fam7—15 (4)	N/A^a^	1.5	0.29	0.32
Normal range (2)^b^	4.24–10.51	4.24–10.51	0.14–1.23	0.48–1.68
Normal range (4–5)^b^	4.63–12.36	4.63–12.36	0.25–1.54	0.43–1.96
Normal range (9–10)^b^	6.08–15.72	6.08–15.72	0.45–2.36	0.52–2.42
Normal range (adults)^b^	6.39–13.49	6.39–13.49	0.7–3.12	0.56–3.52

*N/A, not available*.

*^a^Patient did not receive immunoglobulin substitution*.

*^b^Reference values according to Jolliff et al. (27)*.

*Underlined and italic numbers mean reduced or increased values, respectively*.

Production of various cytokines was impaired in ICOS-deficient patients. In family 5, IL-10, IL-17, IL-4, IL-5, as well as TNFα, TNFβ and IFN-γ were decreased (7). In families 1 and 2, IL-10 and IL-17 secretion were also notably impaired, whereas IFN-γ and IL-13 secretion were normal (19).

### Treatment

**Figure 2:**
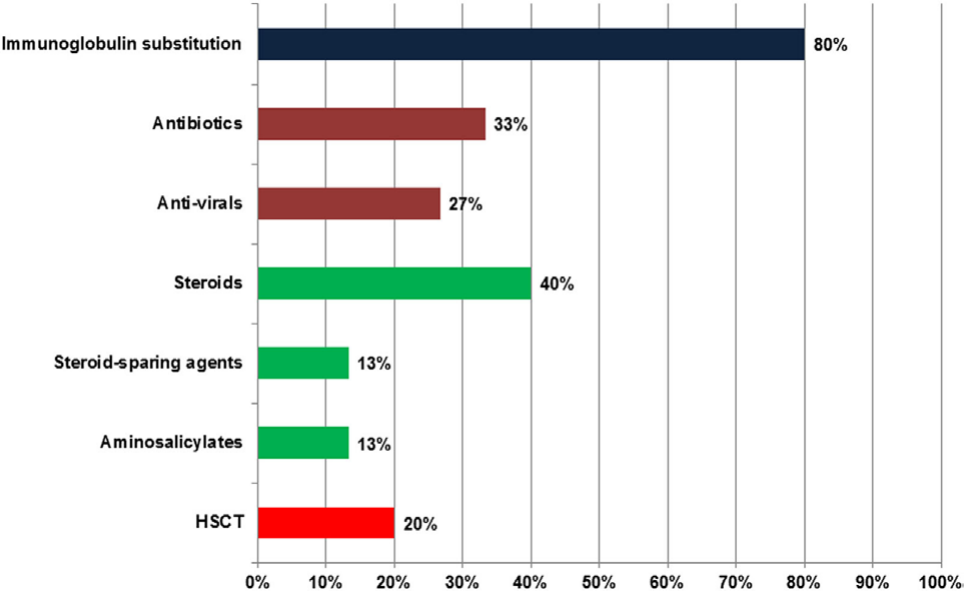
Treatment of inducible co-stimulator (ICOS) deficiency. Total number of patients: 15. The deep blue bar indicates immunoglobulin substitution, green bars show antibiotics and anti-virals, deep red bars represent immune modulatory drugs, and the bright red bar highlights hematopoietic stem cell transplantation (HSCT).

Figure 2 gives an overview on the drugs used for the treatment of ICOS-deficient patients. Eighty percent (12/15) of the patients received immunoglobulin substitution therapy. Eight patients were treated with subcutaneous immunoglobulins, one only during the winter months. Four were treated with intravenous immunoglobulins, not including patient #13 who had low normal IgG trough levels. Two additional patients did not receive immunoglobulins; #11 did not have recurrent infections and the parents of #15 refused treatment.

Despite immunoglobulin replacement, 33% (5/15) needed antibiotics. Of those, two patients (#1, #7) received regular antibiotic prophylaxis with norfloxacin and clarithromycin, respectively. Four patients received antiviral drugs. Two patients were treated with valganciclovir for a CMV infection. The HHV6 infection in patient #14 was treated with intravenous ganciclovir followed by oral valganciclovir together with nitazoxanide. Valaciclovir was used in patient #2 for Herpes keratitis.

**Figure 3:**
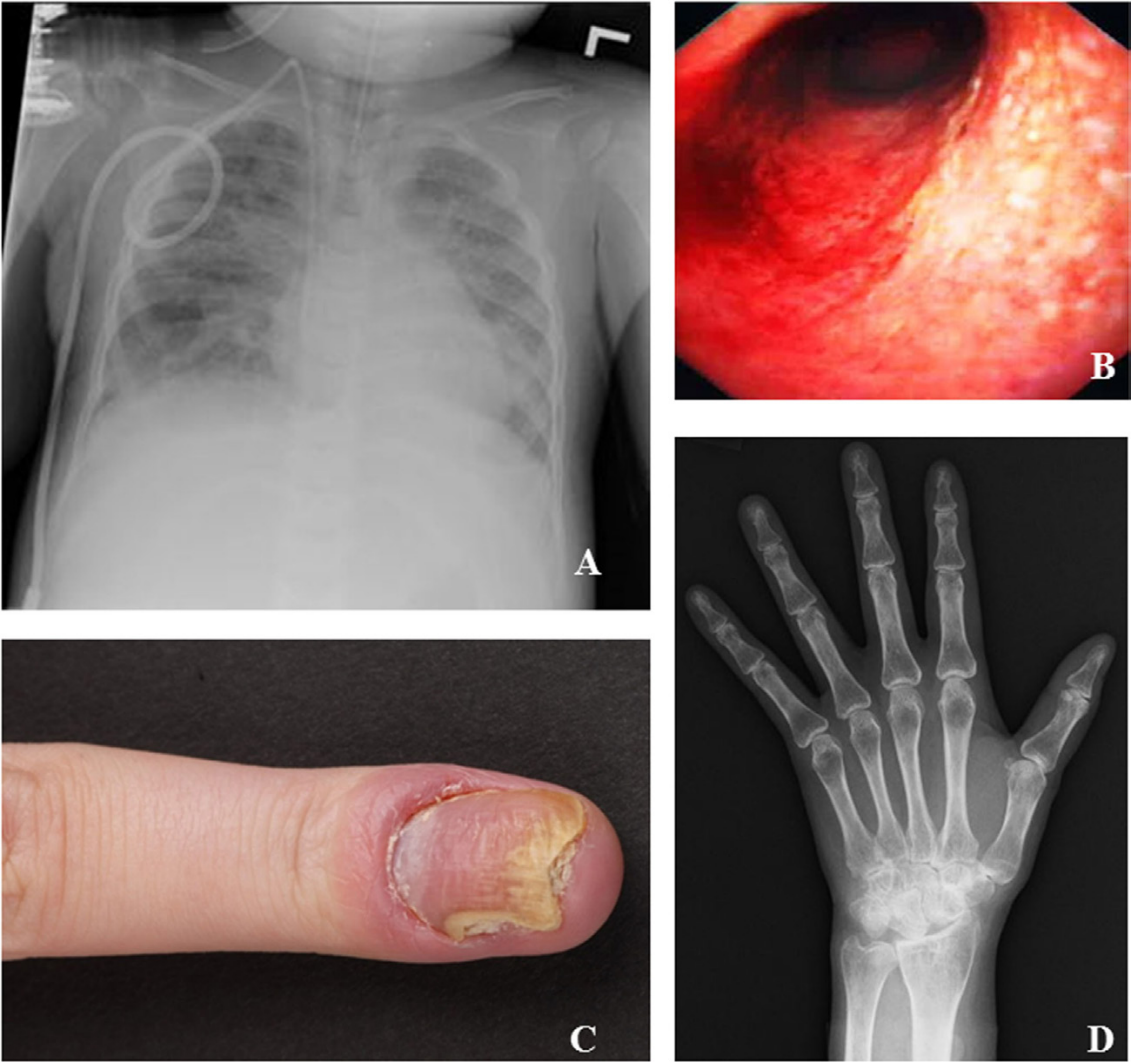
Specific clinical manifestations of inducible co-stimulator (ICOS) deficiency. **(A)** Chest X-ray of patient #12 at the time of respiratory failure shows multifocal patchy consolidative opacities with moderate pulmonary edema. **(B)** Colonoscopy of patient #9 shows inflammatory bowel disease. **(C)** Finger of patient #7 with psoriasis of the nail and secondary *Candida* infection. **(D)** Hand X-ray of patient #10 shows erosive changes suspicious of rheumatoid arthritis.

Corticosteroids were prescribed to 40% (6/15) of patients. Five patients received systemic corticosteroids for the following indications: immune thrombocytopenia and neutropenia, IBD, symmetric rheumatoid arthritis, and herpes keratitis. One patient received intra-articular steroids to treat his ankle joint arthritis. Enteral budesonide was used in one patient with IBD. Additional steroid-sparing agents were necessary in two patients: #6 received cyclosporine for T-cell mediated neutropenia, and #10 received methotrexate for rheumatoid arthritis (Figure 3). Two patients with IBD (Figure 3) improved with mesalazine. In patient #7, antihistamines, antipsoriatic, and antimycotic drugs were applied as topical treatment of her psoriatic nail disease (Figure 3).

Three ICOS-deficient patients have undergone HSCT. One patient (#12) received a matched, related donor transplant at age 2 following a severe *Pneumocystis jirovecii* infection leading to respiratory failure (Figure 3). His symptoms have improved significantly and he is doing well 2 years posttransplant. One patient (#15) suffered from severe colitis and received an unrelated 11/12 human leukocyte antigen matched transplant following reduced intensity conditioning at age 6. However, she developed a capillary leak syndrome on day 5 posttransplant with respiratory distress followed by toxic epidermal necrolysis, and died. The last patient (#6) was transplanted seven months ago at age 53 for persistent agranulocytosis. After reduced intensity conditioning, he received a matched unrelated donor transplant. On day 30, complete donor chimerism was confirmed alongside a complete engraftment. 233 days posttransplantation he showed no additional major complications and no signs of GvHD.

### Genetics

**Figure 4:**
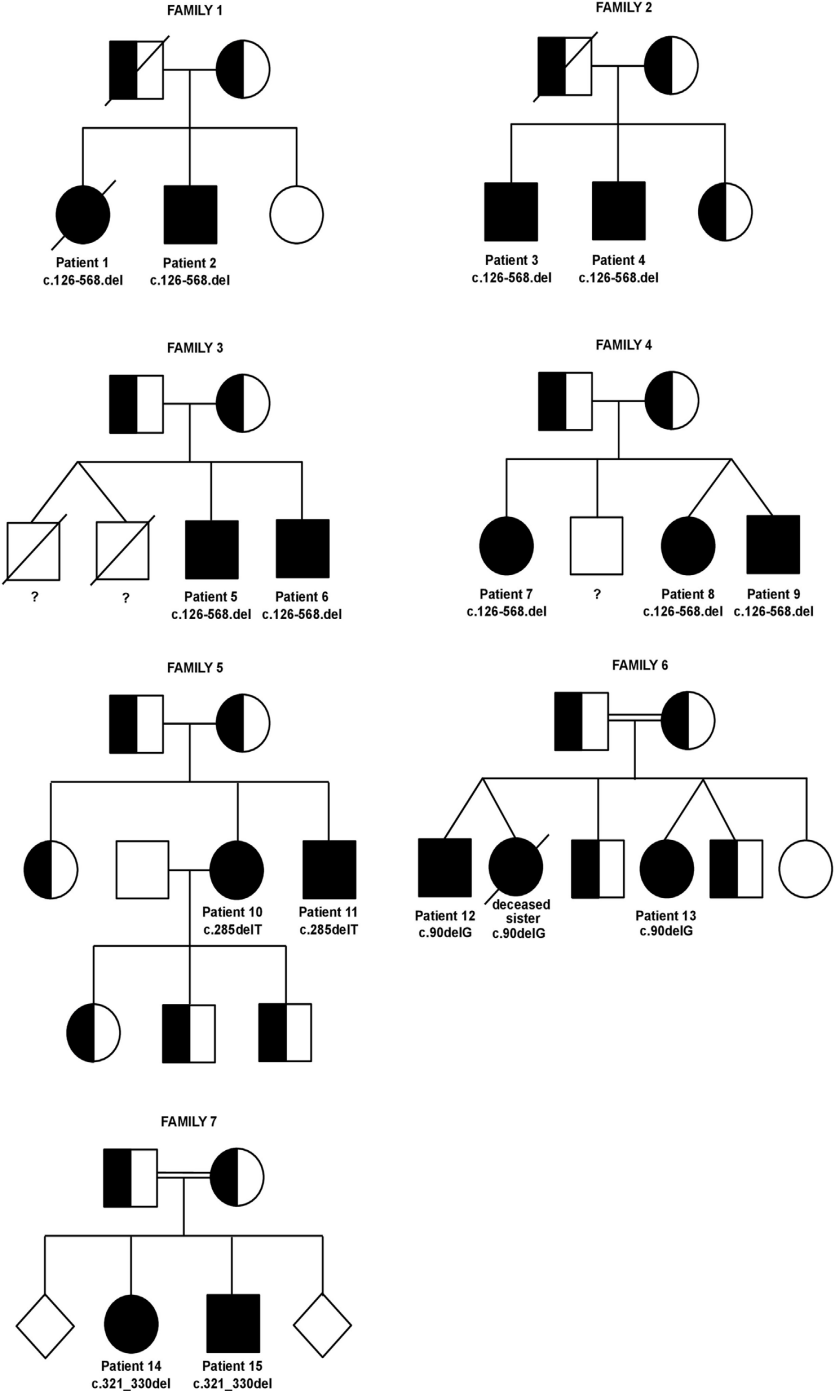
Pedigrees of inducible co-stimulator (ICOS)-deficient families. Twins are joined by diagonal lines. Healthy siblings of family 7 are depicted as gender-neutral rhomb symbols to protect the family’s privacy. Specific *ICOS* mutations and patient numbers are depicted underneath the affected homozygous family members. The genotype of the deceased male twins in family 3 as well as of the healthy male sibling in family 4 is unknown (indicated by a question mark underneath the symbols).

Figure 4 gives an overview on the seven families’ pedigrees. The inheritance was strictly autosomal-recessive in all families, and all mutations led to a failure to detect the protein on the surface of activated T-cells. Families 1–4 had a homozygous deletion of a 1,815 base pair region including exon 2, intron 2, exon 3, and parts of intron 3 of the *ICOS* gene (c.126-568.del) (5, 6). This out of frame deletion led to a premature stop codon and shortened transcript (5). A common ancestor is assumed for these four families. In the unrelated family 5, sequencing of the *ICOS* gene revealed a homozygous deletion of a T at codon 285, leading to a frameshift and the introduction of a stop codon at amino acid position 121 and resulting in a truncated *ICOS* transcript (c.285delT) (7). The patients from family 6 from Kuwait were found to carry a single nucleotide homozygous deletion in Exon 2 (c.90delG), leading to a frameshift and premature stop codon in Exon 2 (8). In the children of the consanguineous family 7, a homozygous 10 base-pair deletion in *ICOS* (c.321_330del) led to a frameshift and a premature stop after 10 codons in the new reading frame (p.F108YfsX118) (9).

## Discussion

These 15 ICOS-deficient patients teach various lessons about the role of ICOS: All reported *ICOS* mutations were deletions, resulting in an undetectable ICOS protein. Despite this uniform effect on protein expression, the clinical presentations varied. The onset of disease was very variable in the cohort. As patient #8 still did not display any symptoms at age 15 in contrast to her dizygotic twin brother, her condition will be watched closely. In case she develops symptoms, this might give new insights into triggering events.

Inducible co-stimulator deficiency clearly has its major impact on the maintenance of GCs and, therefore, on the normal development of class switched B-cell responses. This resulted in hypogammaglobulinemia in all but one patient, leading to increased susceptibility to bacterial infections in most individuals. In some patients with ICOS deficiency, the pattern of viral susceptibility rather suggests a combined immunodeficiency (CID). There were recurrent Herpes infections in families 1, 3, and 4 (5, 19), and prolonged viral infections in childhood in family 5 (7). This is also relevant for the potential role of ICOS in tumor surveillance for virally induced tumors, highlighted by the HPV-associated vulvar carcinoma in patient #1 and the squamous cell carcinoma in patient #6. The latter is supported by data demonstrating that ICOS stimulation enhances NK-mediated tumor cell killing in mice (28). Also, impaired T-cell activation was reported in ICOS knockout mice (29).

Intriguingly, in the recently reported families 6 and 7, the occurrence of *Pneumocystis jirovecii* pneumonia, defective handling of HHV6 and possibly also of *Cryptosporidium*, were associated with a very early onset of disease (8, 9). None of the other ICOS-deficient patients had frankly opportunistic infections or showed signs of a severe T-cell deficiency. Even in the patients from family 5 who showed deviations in the T-cell memory compartment, no opportunistic infections were observed. It remains to be clarified whether this is a variation of the clinical presentation of ICOS deficiency or if additional modifiers are involved.

Additionally to the infectious profile, the increased prevalence of immune dysregulation (77%) also suggests an underlying CID. This is highlighted by the patients’ susceptibility to psoriasis (4/15 patients) and enteropathy (9/15 patients). Immune dysregulation in ICOS deficiency seems less pronounced than in LRBA deficiency [in which 95% of patients are affected (30)]. The prevalence of immune dysregulation in CVID seems to depend on the cohort. Gathmann et al. found 29% of the patients suffering from autoimmunity and 9% from enteropathy (2), whereas Cunningham-Rundles and Bodian found 22% of autoimmunity and 6% of IBD (3). Several observations hint toward a role of ICOS-mediated signaling in autoimmunity. For example, it has been shown that plasmacytoid dendritic cells prime IL-10-producing T regulatory cells *via* ICOS-L (31). Yet, the precise autoimmune context of ICOS triggering on T-cells and the consecutive T-cell help to the antigen presenting bystander cells has not yet been defined.

Several abnormalities of the B- and the T-cell compartment should be highlighted: In ICOS deficiency, there seems to be a progressive loss of B-cells, reflected by a normal B-cell count in childhood but impaired counts in all but two adult patients, including those followed longitudinally. In several ICOS-deficient patients, a block in B-cell development after the pre-BI stage was reported. This developmental block is not ICOS-specific, as it was also detected in ICOS-positive CVID-like patients (32), and it is reminiscent of the severe block seen in Bruton’s tyrosine kinase-deficiency (33). Also, naïve B-cell counts were reduced in most, but not all ICOS-deficient patients. This might hint toward a progressive bone marrow output failure. Switched memory B-cells are reduced, but still detectable at varying levels in all ICOS-deficient patients. Their severe reduction is in part explained by the crucial role of ICOS:ICOS-L interactions in the GC; however, B-cell differentiation seemingly does not totally depend on this support.

Regarding the T-cell compartment, the decreased proportion of memory T-cells that were found in several ICOS-deficient individuals could be a specific finding of this condition: ICOS was shown to control the pool size of TEMs (34). Nevertheless, this phenomenon was not present in the entire cohort, and the CD8 effector memory cells were even high in one patient (patient #12), again possibly due to chronic infection by *Candida*, CMV and *Pneumocystis jirovecii*. In contrast, the reduction of circulating CXCR5^+^CD45RO^+^ CD4 TFH-cells was reported in all ICOS-deficient patients and coincides with an impaired GC formation in lymphoid tissues as it has been observed also in CD40L deficiency (35). This accords with a role for ICOS as a crucial survival signal for CXCR5^+^ TFH-cells (20). ICOS^high^ T-cells have been shown to produce IL-10 (36), and maintain the pool size of FOXP3^+^ Tregs (34). These suggestions from the mouse model could only be affirmed in the patients of family 5, who showed a reduction of FOXP3 expression together with contraction of the CD45RO^+^CTLA4^+^ Treg subset producing IL10. However, these findings were not confirmed in any of the other ICOS patients who were evaluated for FOXP3 expression, suggesting a less stringent need for human ICOS expression in the development or maintenance of these cell types.

The patients’ interleukin-profile also varied substantially in between the reported patients, but, it is striking that in all patients who were assessed for cytokine expression profiles, IL-17 was markedly decreased. Pathogenic mutations of several genes can impair TH17 development, including STAT1, STAT3, DOCK8, AIRE, and IL-17RA, or IL-17F (37). Most of the associated conditions confer a risk of (invasive) *Candida* infections, as was also observed in patient #12, who suffered from *Candida*-induced acute respiratory failure at 1 month of age. Otherwise, there were only occasional local super-infections in patients with psoriasis (e.g., patient #7, Figure 3). *P. jirovecii* pneumonia (patient #12), CMV infections (patients #7, #9, #10, and #12) and *S. aureus* skin infections (patient #1) have been associated with impaired TH17 development. In the two siblings of family 5, the production of IL-22 was tested additionally and found to be normal. IL-22 is another Th17 cytokine that is crucial in the pathogenesis of psoriasis and both patients had psoriasis (as well as patient #7 and #9 from family 4). While it remains unclear whether IL-17A and IL-22 are produced by the same Th17 subset (38, 39), these data suggest that an IL-22-producing subset is not impaired in ICOS deficiency.

The spectrum of clinical manifestations led to a broad spectrum of treatments. Almost all ICOS-deficient patients were on immunoglobulin replacement therapy. Corticosteroids were also used frequently, and two patients needed additional steroid-sparing agents. Most ICOS-deficient patients could be managed with conservative therapy. This is especially true for patients with a late onset of disease and the main sequelae being hypogammaglobulinemia. However, three ICOS-deficient patients received HSCT due to CID (#12), persistent agranulocytosis (#6) or severe colitis (#15); the latter died following posttransplant complications. Early transplantation before accumulation of disease complications and acquisition of viral infections may improve outcome in a subset of patients with significant T-cell dysfunction, autoimmunity, life-threatening infections or severe cytopenias.

The clinical and laboratory data from these 15 ICOS-deficient patients, indicate that more emphasis should be placed on the T-cell dysregulation in this disorder. In 2013, ICOS deficiency was classified as “Predominantly antibody deficiency“ by the International Union of Immunological Societies Expert Committee on Primary Immunodeficiency (40), but in 2015, it was reclassified as CID (41), describing the clinical phenotype with the key words “autoimmunity, gastroenteritis, may have granuloma.” This classification is certainly supported by our data. In addition, the rare occurrence of opportunistic infections should be considered as possible alteration of the phenotype. CD40L deficiency is another disease that was first described as a defect of B-cell isotype switching but is now known to be a defect of co-stimulatory T-cell help and function (40). As with CD40L deficiency, ICOS deficiency should be considered in CVID-like disease as well as in CID patients.

## Conclusion

Inducible co-stimulator deficiency has a broad clinical and immunological phenotype. Whereas in the initially published families, ICOS deficiency seemed to produce isolated hypogammaglobulinemia, the recently published patients showed more complex phenotypes with prominent autoimmune features and opportunistic infection profiles. There is now ample evidence that B-cell counts decline during the course of the disease, possibly related to a progressive bone marrow output failure. Genetic analysis is required to reliably distinguish ICOS deficiency from other immunological defects. ICOS deficiency should be ruled out in patients in whom immunodeficiency and autoimmunity or immune dysregulation coincide.

## Ethics Statement

This study was carried out in accordance with the recommendations of the ethics protocol No.; 295/13 of the Ethik-Kommission des Universitätsklinikums Freiburg with written informed consent from all subjects. All subjects gave written informed consent in accordance with the Declaration of Helsinki. The protocol was approved by the Ethik-Kommission des Universitätsklinikums Freiburg.

## Author Contributions

JS: writing of the manuscript, data collection of all families, and data interpretation of all families. JC: revision of the manuscript, data collection family 6, and data interpretation. AS-B: revision of the manuscript, data collection family 4, and data interpretation. PA, KE and SH: revision of the manuscript, data collection family 7, and data interpretation. TM: revision of the manuscript, data collection family 5, and data interpretation. ER: revision of the manuscript, data collection family 2, and data interpretation. KW and BG: revision of the manuscript, data collection family 1–4, and data interpretation. RG: revision of the manuscript, data collection family 6, and data interpretation.

## Conflict of Interest Statement

The authors declare that the research was conducted in the absence of any commercial or financial relationships that could be construed as a potential conflict of interest.

## Abbreviations

CVID, common variable immunodeficiency; ICOS, inducible co-stimulator; CTLA-4, cytotoxic T-lymphocyte-associated Protein 4; PD1, programmed cell death protein 1; Erk1/2, extracellular signal-regulated kinase 1/2; PKB, protein kinase B; PDK1, Phosphoinositide-dependent kinase-1; PI3K, phosphatidylinositol-3-kinase; IL, interleukin; GM-CSF, granulocyte-macrophage colony-stimulating factor; IFN-γ, Interferon gamma; Tregs, regulatory T-cells; GC, germinal center; CXCR5, C-C chemokine receptor type 5; TFH, follicular T-helper-cells; CXCL13, chemokine (C-X-C motif) ligand 13; FDC, follicular dendritic cells; HPV, human papilloma virus; Ig, immunoglobulin; CMV, cytomegalovirus; ITP, idiopathic thrombocytopenic purpura; LGL T-cells, T-cell large granular lymphocyte cells; SCIG, subcutaneous immunoglobulin; IVIG, intravenous immunoglobulin; IBD, inflammatory bowel disease; HSCT, hematopoietic stem cell transplantation; HLA, human leukocyte antigen; TEM, effector memory T-cell; TCM, central memory T-cell; FoxP3, Forkhead box P; TGF-β, transforming growth factor beta; HHV6, human herpesvirus 6; Btk, Bruton’s tyrosine kinase; CID, combined immunodeficiency.
