# Supplementary material for: 14 Years after Discovery: Clinical Follow-up on 15 Patients with Inducible Co-Stimulator Deficiency
Source: Front Immunol. 2017 Aug 16;8:964. doi: 10.3389/fimmu.2017.00964 (PMC5561331; doi:10.3389/fimmu.2017.00964)
Supplement: Supplementary file 1 [file table_1.pdf]

| Patient ID | Fam1-01 | Fam1-02 | Fam2-03 | Fam2-04 | Fam3-05 | Fam3-06 | Fam4-07 | Fam4-08 | Fam4-09 | Fam5-10 | Fam5-11 | Fam6-12 | Fam6-13 | Fam7-14 | Fam7-15 |
|---|---|---|---|---|---|---|---|---|---|---|---|---|---|---|---|
| Age at evaluation | 44 | 46 | 47 | 37 | 56 | 52 | 28 | 15 | 15 | 47 | 44 | 3 | 0 | 5 | 4 |
| Age of onset | 27 | 19 | 28 | 19 | 15 | 12 | 8 | - | 1 | 35 | 35 | 0.5 | 3 | 2 | 2 |
| Sex | female | male | male | male | male | male | female | female | male | female | male | male | female | female | male |
| Deceased | + | - | - | - | - | - | - | - | - | - | - | - | - | + | - |
| Consanguinity | - | - | - | - | - | - | - | - | - | - | - | + | + | + | + |
| **Recurring infections** | + | + | + | + | + | + | + | - | + | + | - | + | - | + | - |
| Lower respiratory infections | + | + | + | + | + | + | + | - | - | - | - | + | - | + | - |
| Upper respiratory infections | + | + | + | + | + | + | + | - | - | - | - | + | - | + | - |
| Herpes infections | - | + | - | - | + | + | + | - | + | + | - | + | - | + | - |
| Intestinal infections | - | + | - | + | - | - | - | - | - | - | - | - | - | + | - |
| Otitis media | - | - | + | - | - | - | - | - | - | - | - | - | - | - | - |
| Vaginal ulcer | - | - | - | - | - | - | - | - | - | + | - | - | - | - | - |
| Staphylococcal impetigo | + | - | - | - | - | - | - | - | - | - | - | - | - | - | - |
| Verrucosis | - | - | - | - | - | + | - | - | - | - | - | - | - | - | - |
| Bacteremia and sepsis | - | - | - | - | - | - | - | - | - | + | - | - | - | - | - |
| Lymphocytic meningitis | - | - | - | - | + | - | - | - | - | - | - | - | - | - | - |
| **Opportunistic Infections** | - | - | - | - | - | - | - | - | - | - | - | + | - | - | - |
| **Autoimmunity/ Immune dysregulation** | - | + | - | + | + | + | + | - | + | + | + | + | + | + | - |
| Enteropathy | - | + | - | + | - | - | + | - | + | + | - | + | + | + | + |
| Splenomegaly | - | + | - | + | + | + | - | - | - | - | - | - | - | - | - |
| Psoriasis | - | - | - | - | - | - | + | - | + | + | + | - | - | - | - |
| Arthritis | - | - | - | - | - | - | - | - | + | + | + | - | - | - | - |
| Hepatomegaly | - | - | - | + | - | + | - | - | - | - | - | - | - | + | - |
| Non-infectious hepatitis | + | - | - | + | - | - | - | - | - | - | - | - | - | - | + |
| Thrombocytopenia | - | - | - | - | + | - | - | - | - | + | - | - | - | - | - |
| Neutropenia | - | - | - | - | + | + | - | - | - | - | - | - | - | - | - |
| Granulomas | - | + | - | + | - | - | - | - | - | - | - | - | - | - | - |
| Eczema | - | + | - | - | + | - | - | - | - | - | - | - | - | - | - |
| Interstitial pneumonitis | - | - | - | - | - | - | - | - | - | + | - | - | - | - | - |
| **Malignancy** | + | - | - | - | - | + | - | - | - | - | - | - | - | - | - |
| **Neurologic/psychiatric** | - | - | - | - | + | - | - | - | - | - | - | - | - | - | - |
| **Treatment** | + | + | + | + | + | + | + | + | + | + | + | + | - | + | - |
| Immunoglobulin substitution | SCIG | SCIG | SCIG | SCIG | SCIG | SCIG | IVIG | SCIG | SCIG | IVIG | - | IVIG | - | IVIG | - |
| Steroids | - | + | - | - | - | + | + | - | + | + | + | - | - | - | - |
| Immunosuppressants | - | - | - | - | - | + | - | - | - | + | - | - | - | - | - |
| Aminosalicylates | - | - | - | - | - | - | + | - | - | + | - | - | - | - | - |
| Antibiotics | + | - | + | - | - | - | + | - | - | + | + | - | - | - | - |
| Anti-virals | - | + | - | - | - | - | + | - | + | - | - | - | - | + | - |
| HSCT | - | - | - | - | - | + | - | - | - | - | - | + | - | + | - |
| Antimycotics | - | - | - | - | - | - | + | - | - | - | - | - | - | - | - |
| Antipsoriatics | - | - | - | - | - | - | + | - | - | - | - | - | - | - | - |
| Antihistamines | - | - | - | - | - | - | + | - | - | - | - | - | - | - | - |

SCIG, subcutaneous immunoglobulin; IVIG, intravenous immunoglobulin; HSCT, hematopoietic stem cell transplantation
