# Supplementary material for: 14 Years after Discovery: Clinical Follow-up on 15 Patients with Inducible Co-Stimulator Deficiency
Source: Front Immunol. 2017 Aug 16;8:964. doi: 10.3389/fimmu.2017.00964 (PMC5561331; doi:10.3389/fimmu.2017.00964)
Supplement: Supplementary file 2 [file table_2.pdf]

**Patient ID/ Ref. Values (years)**	**Fam1- 01**	**Fam1- 02**	**Fam2- 03**	**Fam2-04**	**Fam3-05**	**Fam3- 06***	**Fam4-07**	**Fam4-08**	**Fam4-09**	**Fam5-10**	**Fam5-11**	**Fam6-12***	**Fam6-12#**	**Fam7-15**	**Fam7-16**	**Ref. Values**	**Ref. Values**	**Ref. Values**	**Ref. Values**
**Age at evaluation (years)**	42	43	46	34	55	52*	28	15	15	48	46	2*	3#	6	8				
Hemoglobulin (g/dL)	14,4	16,3	14,8	12,9	14,8	13,3	11,8	11,6	10,4	9,8	16,5	8	*13,2*	13,2	13,7	11.0-12.9	11.0-13.4	11.0-14.3	14-18
Platelets (10^3cells/µL)	116	181	236	103	107	67	234	269	297	97	188	111	232	213	271	190-370	219-350	180-307	140-400
White Blood cells (10^3cells/µL)	6,5	6,5	6,8	8,57	0,5	0,6	8,2	8,35	9,48	5,5	5,9	4,47	9,7	8,3	9,3	6.0-10.8	5.4-9.9	5.2-9.7	04. Okt
Neutrophils (10^3cells/µL)	4,4	4,5	4,58	*6,8*	0,3	0,02	5,5	4,8	6,4	3,62	4,07	1,79	4,78	4,46	4,88	2.3-6.4	2.6-6.3	2.7-6.7	1.8 - 6.2
Monocytes (10^3cells/µL)	0,48	0,73	*1,2*	0,7	N/A	N/A	0,3	0,6	0,7	0,3	0,4	0,18	0,39	0,6	*1,1*	0.3-1.2	0.3-0.9	0.4-1.3	0.26 - 0.87
Lymphocytes´(10^3cells/µL)	1,3	0,8	0,6	0,9	0,6	0,27	2	2,1	2,2	1,36	1,35	2,36	4,15	3,18	3,15	1.3-3.8	1.2-2.8	1.0-2.3	1.0-2.8
**Age at evaluation (years)**	42	43	36	24	55	52	27	15	15	48	46	2	3	3	5				
**T cells(CD3+) cells/µL (% of lymphocytes)**	1.2 (*95*)	0.7 (*92*)	0.9 (78)	0.3 (*92*)	0.5 (81)	0.25 (*95*)	1.2 (60)	1.2 (59)	1.3 (61)	1.06 (78)	1.2 (*91.4*)	0.3 (*81*)	0.4 (69)	3.2 (69)	2.9 (50)	0.9-4.5 (43-76)	0.7-4.2 (55-78)	0.8-3.5 (52-78)	0.7-2.1 (55-83)
CD3+CD4+, cells/µL (% of CD3+)	0.7 (59)	0.3 (44)	0.6 (50)	0.2 (44.8)	0.3 (55)	0.009 (35)	0.5 (*41*)	0.7 (*55*)	0.7 (*53*)	1.0 (*73*)	0.9 (*68*)	0.1 (45)	0.9 (17)	2.4 (*52*)	1.9 (32)	0.5-2.4 (23-48)	0.3-2.0 (27-53)	0.4-2.1 (25-48)	0.3-1.4 (28-57)
CD3+CD8+, cells/µL (% of CD3+)	0.4 (*32*)	0.3 (*45*)	0.3 (23)	0.1 (*33.6*)	0.1 (17)	0.14 (*55*)	0.7 (*56*)	0.5 (*44*)	0.6 (*45*)	0.3 (25)	0.4 (29)	0.1 (34)	0.3 (*49*)	0.6 (13)	0.9 (15)	0.2-0.9 (14-33)	0.3-1.8 (19-34)	0.2-1.2 (9–35)	0.2-0.9 (10-39)
**B cells (CD19+) cells/µL (% of lymphocytes)**	0.009 (0.7)	0.009 (1.2)	0.03 (2.9)	0.005 (1.4)	0.07 (12)	0.006 (2)	0.01 (0.65)	0.1 (5.8)	0.06 (2.8)	0.01 (0.6)	0.06 (4.8)	0.5 (15)	1.3 (25)	1.4 (30)	*2.8 (47)*	0.2-2.1 (14-44)	0.2-1.6 (10-31)	0.2-0.6 (8–24)	0.1-0.5 (6-19)
CD19+CD27-IgD+ (naïve) % of B cells	*95,5*	*88,4*	*93,5*	*87,2*	*95,8*	63,85	42	*92,7*	*98,82*	64,7	*88*	*98,8*	*94,2*	*91*	*97*	76.3–90.1	69.4-80.4	75.2–86.7	58.0–72.1
CD19+CD27+IgD+ (nsm) % of B cells	5,9	8,2	5,5	11	3,6	*30,37*	*45,26*	0	0,3	22	7,3	0,8	1,8	4	3	4.1-9.0	7.5-12.4	4.6–10.2	13.4–21.4
CD19+CD27+IgD- (sm)% of B cells	0,8	1,6	0,3	0,9	0,08	2,1	2,11	1,02	0	6,4	2,5	0,2	3,1	<2	0	1.5–7.4	5.2-12.1	3.3–9.6	9.2–18.9
Plasmablasts % of B cells	N/A	0,3	N/A	N/A	0,01	0,5	0	0,15	0	3,2	0,5	0,2	1,4	N/A	N/A	0.6–2.7	0.7-3.5	0.3–1.7	0.6–1.6
Transitional % of B cells	N/A	1,6	3,7	N/A	*5*	0,75	1,18	1,54	3,29	*24,6*	1,2	0,2	8,5	N/A	N/A	5.1–10.7	4.5-9.2	3.9–7.8	1.0–3.6
**NK cells (CD16+CD56+) cells/µL (% of lymphocytes)**	0.04 (3)	0.05 (7)	0.2 (19)	0.03 (7)	0.03 (5.6)	0.007 (2.5)	0.147 (7)	0.163 (8)	0.08 (3.5)	0.19 (14.3)	0.03 (2.5)	0.08 (3)	0.36 (7)	0.19 (6)	0.06 (3)	0.1-1.0 (4-23)	0.09-0.9 (4-26)	0.07-1.2 (6-27)	0.09-0.6 (7-31)

§ Reference values for pediatric hematology: Reference for Pediatric Values: *Pediatric Reference Intervals,* Fifth Ed, AACC Press, 2005,
Reference values for adult hematology: Central laboratory, University Clinic Freiburg, internal reference ranges
Reference values for lymphocyte subsets: Comans-Bitter et al, J Pediatr. 1997,
Reference values for B cell subsets: Morbach et al, Clin Exp Immunol. 2010,
underlined and *italic* numbers mean reduced or increased values, respectively; *pre transplantation; # post transplantation; data of patient Fam06-13 was not available
