# Supplementary material for: 14 Years after Discovery: Clinical Follow-up on 15 Patients with Inducible Co-Stimulator Deficiency
Source: Front Immunol. 2017 Aug 16;8:964. doi: 10.3389/fimmu.2017.00964 (PMC5561331; doi:10.3389/fimmu.2017.00964)
Supplement: Supplementary file 3 [file data_sheet_1.docx]

**14 years after discovery: Clinical follow-up on 15 patients with ICOS deficiency**

**Supplement**

Johanna Schepp, Janet Chou, Andrea Skrabl-Baumgartner, Peter D. Arkwright, Karin R. Engelhardt, Sophie Hambleton, Tomohiro Morio, Ekkehard Röther, Klaus Warnatz, Raif Geha, Bodo Grimbacher

**Case reports of the ICOS-deficient patients**

**Family 1**

Patient 1 (female) was diagnosed with CVID at age 36 after a period of recurring upper and lower respiratory tract infections, including pneumonia. She had low immunoglobulin levels (IgM, IgG and IgA), and also suffered from recurrent staphylococcal impetigo. Four years later, the patient and her brother (patient 2) were the first to be diagnosed with ICOS deficiency in 2001. At age 34, she had developed a verrucous squamous epidermal cell carcinoma of the vulva secondary to human papilloma virus infection. Her recurrent respiratory tract infections were treated successfully with immunoglobulin substitution later on. During the course of her disease, she developed a chronic steatohepatitis. From age 39, she had recurrent episodes of urticarial exanthema and recurrent staphylodermatitis. She also suffered from impaired wound healing after surgery and radiation of her carcinoma, of which she died at age 44. Histological findings revealed a reduced perilesional lymphocytic infiltrate of the tumor, especially a lack of CD4+ T-cells in comparison to the great abundance of CD4+ T-cells in the ICOS+ control [E^[[1]](#endnote-1)^]. As no immunosuppressive therapy had been given to the patient, these findings suggest a potential role of ICOS in surveillance of virus associated tumors.

Patient 2 (male), the younger brother of patient 1, was diagnosed with CVID at age 21, following a 2 year episode of recurrent upper and lower respiratory tract infections. He was placed on IgG substitution the following year and had since IgG trough levels of about 7g/l. During the course of disease he developed giardiasis at age 23, salmonellosis at age 27, *Campylobacter* enteritis with nodular lymphoid hyperplasia of the intestinal mucosa at age 40, and pneumonia at age 48. His recurrent *herpes simplex* keratitis was treated successfully by keratoplastic of his right eye at age 48, followed by valaciclovir and corticosteroid prophylaxis for one year. Also, low-grade chronic gastritis as well as low-grade colitis were diagnosed at age 30. Due to the lack of distortion of the crypts, an infectious origin of the disease was considered, but a disease-associated pathogen was never identified. His colitis has not changed significantly since first being diagnosed. His initial splenomegaly resolved spontaneously and is no longer detectable. Moreover, the patient has notably improved in frequency and severity of his infections under SCIG treatment.

**Family 2**

Apart from two pneumonias at age 6, patient 3 (male) had a normal frequency of infections until age 28 when a period of recurrent upper respiratory tract infections started. At age 31, he was diagnosed with CVID and put on IgG substitution therapy, first 25 g every 3 or 4 months i.v., followed by 1,6 g s.c. three times a week. At the time of diagnosis, he had already developed bronchiectasis. Even though the patient did not adhere to the IgG substitution with trough levels around 3 g/l, only occasional respiratory infections have occurred during the last decade. At age 39, he suffered from a purulent middle ear infection. Surgery was performed on both ears following perforation. Since age 45, he has not reported any severe infections and is still on IgG substitution as the only therapy. Under this regimen, he does not have any symptoms; no diarrhea, no infections, no autoimmunity. Recently, he has shown first signs of Dupuytren.

Patient 4 (male) is the younger brother of patient 3. He suffered from pneumonia in childhood (age 12). However, he was only diagnosed with CVID at age 22 after three years of recurrent infections including pneumonia and frequent diarrhea. At this time, he was also suffering from recurrent sinusitis and *Salmonella* enteritis. He was placed on intravenous Ig replacement therapy. In the years following his diagnosis, he developed a chronic granulomatous inflammation of the skin with sarcoid-like lesions on his trunk. At age 27, elevated γ-GT and LDH levels, a mild hepatomegaly, and liver fibrosis were reported upon ultrasound. When he was examined at age 34 because of severe upper abdominal pain, these findings were confirmed alongside splenomegaly (15.5 x 7cm). The pathogenesis of his hepatitis remained unclear. Drug-induced toxicity was suspected according to histological finding and intake of a gyrase-inhibitor antibiotic shortly before There was no evidence of autoimmune hepatitis, and sarcoidosis was excluded histologically. In addition, his trough levels still were at 1.8g/l of IgG despite SCIG being prescribed. The patient died at age 37 due to complications of an unrelated condition.

**Family 3**

The family’s history is notable for two twin brothers that died after birth as premature neonates. Following this, two boys were born to the healthy parents: Patient 5, the older son, had recurrent bronchitis and sinusitis in his adolescence. He was diagnosed with CVID at age 20. After diagnosis, he kept suffering from recurrent upper respiratory tract infections, often associated with labial *Herpes simplex* eruptions. He also had a series of pneumonias at age 27, 31 and 38. Recently, a CT scan showed mild emphysema. He developed splenomegaly and mild thrombocytopenia (107.000/µl). Starting age 40, he suffered from intermittent stress-related urticaria. Also, he developed neurologic symptoms (vertigo, ataxia, headache) associated with lymphocytic meningoencephalitis at age 46. Findings of *Borrelia burgdorferi*–specific IgM antibodies in the cerebrospinal fluid suggested Borreliosis of the central nervous system. He was treated with ceftriaxone. Also, IVIG replacement was started and he improved clinically. However, bilateral hypoacusis remained. He had already suffered from mild neutropenia for several years when, at age 54, he developed a bout of severe neutropenia (200/µl). No auto-antibodies were detected, and his CMV and EBV PCR were negative. As a result, he is now being treated with G-CSF from day three onwards of a neutrophil crisis or an infection with fever. Bone marrow analysis at age 40, 46 and 54 showed no evidence of dysplasia or malignant infiltration. He developed a severe infusion reaction to IVIG at age 29; since then his Ig substitution therapy was irregular. He is currently being treated with SCIG.

Patient 6, the youngest child of the family, initially had a phenotype resembling his brother’s. After his first pneumonia at age 12, recurrent respiratory tract infections and localized *Herpes simplex* eruptions on his lips started in adolescence. CVID diagnosis was confirmed at age 15. Since diagnosis, he has always been on Ig substitution treatment, reaching IgG trough levels of > 7 g/l (age 51). The respiratory tract infections are mostly controlled under the SCIG replacement therapy. He is borderline positive for EBV in PCR (500 copies/ml) and suffers from severe generalized verrucosis. At age 53, a pre-auricular squamous cell carcinoma grade I was excised *in toto*. Photodynamic therapy is currently discussed, as all topical therapies have not improved his condition. His most serious complication however, is his autoimmune steroid-dependent neutropenia that was first diagnosed at age 41 following laboratory work-up of his hepatosplenomegaly. Back then, low levels of anti-neutrophil-antibodies were detected, but were never confirmed subsequently. He developed mouth ulcers and gingival inflammations improved following the treatment of his neutropenia with oral steroids and cyclosporine. At age 51, he had a severe bout of neutropenia (500/µl). A bone marrow analysis showed LGL T-cells with clonal expansion. In the peripheral blood, a distinct expansion of double negative T-cells, and CD8+ effector T-cells with high activation markers were noted alongside a profound decrease of CD4+ T-cells (90/µL; NR: 300 – 1400/µL), CD8+ T-cells (147/µL; NR: 200 – 900/µL), NK-cells (7/µL; NR: 90- 600/µL) and ongoing neutropenia (<500/µL; NR: 1800 – 6200/µL) and B-cell lymphopenia (6/µL; NR: 100-500/µL). He has been treated with G-CSF as standby and antibiotic treatment when needed. At age 53, he received an allogeneic stem-cell transplantation for his progressive pancytopenia with recurrent bouts of fever. After reduced intensity conditioning (Thiotepa, Fludarabine and Melfalan), he received a matched unrelated donor transplant. For GvHD prophylaxis, he was treated with ATG, cyclosporin A and mycophenolate mofetil. He developed a bilateral pneumonia during the post-transplant period responding to antibiotic treatment, but leukocytes were >1,000/µL from day 23. On day 30 a complete chimerism was confirmed alongside a complete engraftment. At a clinical follow-up 233 days post-transplantation he showed no additional major complications and no signs of GvHD.

**Family 4**

Unlike patients from families 1 - 3, patients from family 4 developed their first symptoms already during childhood. Patient 7, the eldest daughter, had her first pneumonia at age 8, but – even though shown to be hypogammaglobulinemic at the time – was diagnosed with CVID only at age 15. In the meantime, she had already suffered from various relapses of pneumonia, which eventually destroyed her left lower lobe in spite of antibiotic prophylaxis, resulting in lobectomy at age 12. Several pulmonary infections followed and led to bronchiectasis until she was finally diagnosed with CVID. Since age 15, she is treated with IVIG (currently 30g/every 4 weeks), and her respiratory inflammations are under control. She also developed psoriasis of her nails and scalp at age 20 and herpes genitalis at age 26. Chronic bowel inflammation has been her main clinical problem since age 26 when the disease manifested during her first pregnancy. She was initially diagnosed with ulcerative colitis (UC), and has been treated accordingly with corticosteroids. At age 27, PCR results from blood, urine and stool showed an active CMV infection that was treated successfully with valganciclovir. Stool frequency and quality are stable now under Mesalazine treatment. She still has a rectovaginal fistula, indicating active bowel inflammation.

Patient 8 (female) and patient 9 (male) are dizygotic twins that carry the identical ICOS mutation. In contrast to her affected siblings, patient 8 (currently age 15) does not have an increased susceptibility to infections, nor skin- or bowel- involvement despite very low IgG levels (3.5g/L). Ig substitution is only carried out during winter months. At age 15, she had elevated calprotectin levels in her stool. However, as she does not complain about any symptoms, her condition is currently watched carefully.

Patient 9 suffered from non-infectious watery and bloody diarrhea at 18 months of age. Colonoscopy demonstrated nodular lymphoid hyperplasia, a phenomenon reported in about 20% to 30% of CVID patients [E2]. Patient 9 was diagnosed with CVID at age three and put on intravenous Ig replacement therapy, which greatly improved his symptoms. Nevertheless, he experienced a paralytic ileus at age 7 during a bout of his chronic IBD-like colitis. Currently, his abdominal symptoms are stable on oral steroid treatment (budesonide). A CMV infection at age 7 has been successfully treated with valganciclovir. He has also developed psoriasis vulgaris at age 10 years, starting as psoriasis inversa in the genital area and later also on the scalp. Symptoms improved under topical treatment including steroids. At age 15, he showed a symmetric swelling of his ankle joints on both sides, and juvenile idiopathic arthritis was suspected according to sonomorphological findings. Autoantibodies were not detected. His symptoms improved with nonsteroidal anti-inflammatory-drugs (Naproxen), as well as with the application of intra-articular steroids (twice within one year).

**Family 5**

Patient 10, the older sister of the Japanese family, suffered from recurrent prolonged viral infections (measles, chicken pox and rubella) in childhood. At age 34, she underwent appendectomy and developed a pulmonary abscess. She was diagnosed with CVID and put on Ig treatment, reaching trough levels of about 4 g/L. Since, infections are well controlled with no need of antibiotic treatment, but she has developed various disease-related complications during the last years: At age 36, she was diagnosed with inflammatory bowel disease following an episode of abdominal colic and diarrhea. At the same time, she developed immune thrombocytopenia, which were both successfully treated by oral steroids. One year later, she had developed psoriasis and symmetric rheumatoid arthritis with proliferative synovitis of multiple finger and toe joints with erosive changes on x-ray examination. Differential diagnosis of psoriatic arthritis was ruled out based on the joints affected and x-ray findings. RF however, is negative. Her clinical condition is now stable on oral steroid treatment (8-9 mg/day) and methotrexate (10mg/week). Since age 44, she suffers from a vaginal ulcer caused by CMV. Also, she has been thrombocytopenic for the last 3 years; platelet counts remain stable in the range of 50.000/µl. At age 49, she had episodes of fever with elevated CRP. A microbiological PCR-survey including 17 viruses, mycobacterium, fungi and bacteria had no positive result. Further investigations revealed a *Helicobacter cinaedi* bacteremia, that was successfully treated with ceftriaxone and minocycline. At age 50, following an *Escherichia coli* urinary tract infection, she developed a septic shock requiring intensive care treatment including noradrenaline, meropenem and ceftriaxone.

Patient 11 is the younger brother of patient 10, and has a less severe form of immunodeficiency. He had slightly low IgG and psoriasis-like lesions at age 35 (at this time his sister was diagnosed with CVID). There was no need for Ig substitution so far, and his skin lesions are well controlled with topical steroid treatment. At age 44, he developed arthralgia of the MCP joint of his fingers, and laboratory findings showed elevated MMP-3 of 118 – 167 ng/mL (normal range <121 ng/mL) which is associated with radiographic progression in RA [E3]. While ANA was negative, his RF levels were 24 – 27 IU/mL (normal range <15IU/mL). Hence, rheumatoid arthritis was suspected, but has not been treated so far. In contrast to his sister, he did not have any signs of enteropathy.

**Family 6**

At one month of age, patient 12 (male) from Kuwait suffered from Candida-induced acute respiratory failure, CMV viremia, and chronic diarrhea, depending on total parenteral nutrition. At 6 months, low IgG and IgA levels (with elevated IgM levels) were detected and he was placed on IVIG. Nevertheless, trough levels of IgG still came down to 1.5g/L at age 2. At this age, he presented with a *Pneumocystis jirovecii* infection resulting in rapidly progressive respiratory failure and intubation. Following matched, related donor hematopoietic stem-cell transplantation (HSCT), his clinical symptoms have improved significantly and he remains well two years post-transplant.

His twin sister had already died of a septic shock in infancy, and little is known about her clinical phenotype. She had also carried the ICOS mutation, but was not taken into account for this study due to the lack of information.

Patient 13, their sister, is also a homozygous mutation carrier and had low IgG levels at three years of age. Reevaluation at age 10 showed low normal IgG trough levels. She did not receive HSCT but still suffers from chronic diarrhea that is currently not treated. She does not have recurrent infections.

**Family 7**

Patient 14 (female) presented at age 2 years with chronic diarrhea associated with abdominal pain, fever, lethargy and weight loss. At age 3.5 years she was diagnosed to have absent class-switched memory B-cells (CD19 + CD27 + IgD-), hypogammaglobulinemia and impaired vaccine responses. After initiation of immunoglobulin replacement therapy, diarrhea still persisted. Single stool samples were positive for norovirus, adenovirus and *Cryptosporidium*, and a gut biopsy revealed severe active chronic panenteritis. She developed hepatomegaly associated with raised liver enzymes (alanine aminotransferase [ALT] of 2907 IU/L, normal range 10 to 40; gamma-glutamyl transferase [GGT] of 152 IU/L, normal range 0–51). A liver biopsy showed mild chronic hepatitis. Human herpesvirus 6 (HHV6) was confirmed in samples from the sigmoid colon, duodenum and liver. She was treated with intravenous ganciclovir and then oral valganciclovir together with nitazoxanide. Despite liver and duodenal samples becoming negative for HHV6, a sigmoid biopsy remained positive and clinically, severe colitis associated with diarrhea and abdominal pain persisted. She also suffered from recurrent respiratory infections including pneumonia. At age 6, her condition led to the decision for hematopoietic stem-cell transplantation (HSCT) with an unrelated 11/12 HLA matched transplant following reduced intensity conditioning. However, she developed a capillary leak syndrome on day 5 post-transplant with respiratory distress followed by toxic epidermal necrolysis, and died.

Patient 15 (male) is the younger brother of patient 14 and presented at age two years with an episode of diarrhea. Investigations again revealed a similar phenotype with low immunoglobulins, absent class-switched memory B-cells and raised liver enzymes. PCR analyses of blood for CMV, EBV, HHV6 and adenovirus were negative. An episode of hepatitis resolved spontaneously and no specific cause could be determined. According to the family’s wish, he is not under any treatment regimen at the moment and remains clinically well at age 7 years.

1. **References:**

   [E1] [Warnatz K](http://www.ncbi.nlm.nih.gov/pubmed?term=Warnatz%20K%5BAuthor%5D&cauthor=true&cauthor_uid=16384931)., [Bossaller L](http://www.ncbi.nlm.nih.gov/pubmed?term=Bossaller%20L%5BAuthor%5D&cauthor=true&cauthor_uid=16384931)., [Salzer U](http://www.ncbi.nlm.nih.gov/pubmed?term=Salzer%20U%5BAuthor%5D&cauthor=true&cauthor_uid=16384931)., [Skrabl-Baumgartner A](http://www.ncbi.nlm.nih.gov/pubmed?term=Skrabl-Baumgartner%20A%5BAuthor%5D&cauthor=true&cauthor_uid=16384931)., [Schwinger W](http://www.ncbi.nlm.nih.gov/pubmed?term=Schwinger%20W%5BAuthor%5D&cauthor=true&cauthor_uid=16384931)., [van der Burg M](http://www.ncbi.nlm.nih.gov/pubmed?term=van%20der%20Burg%20M%5BAuthor%5D&cauthor=true&cauthor_uid=16384931)., [van Dongen J.J](http://www.ncbi.nlm.nih.gov/pubmed?term=van%20Dongen%20JJ%5BAuthor%5D&cauthor=true&cauthor_uid=16384931)., [Orlowska-Volk M](http://www.ncbi.nlm.nih.gov/pubmed?term=Orlowska-Volk%20M%5BAuthor%5D&cauthor=true&cauthor_uid=16384931)., [Knoth R](http://www.ncbi.nlm.nih.gov/pubmed?term=Knoth%20R%5BAuthor%5D&cauthor=true&cauthor_uid=16384931)., [Durandy A](http://www.ncbi.nlm.nih.gov/pubmed?term=Durandy%20A%5BAuthor%5D&cauthor=true&cauthor_uid=16384931)., [Draeger R](http://www.ncbi.nlm.nih.gov/pubmed?term=Draeger%20R%5BAuthor%5D&cauthor=true&cauthor_uid=16384931)., [Schlesier M](http://www.ncbi.nlm.nih.gov/pubmed?term=Schlesier%20M%5BAuthor%5D&cauthor=true&cauthor_uid=16384931)., [Peter H.H](http://www.ncbi.nlm.nih.gov/pubmed?term=Peter%20HH%5BAuthor%5D&cauthor=true&cauthor_uid=16384931)., [Grimbacher B](http://www.ncbi.nlm.nih.gov/pubmed?term=Grimbacher%20B%5BAuthor%5D&cauthor=true&cauthor_uid=16384931). 2006. Human ICOS deficiency abrogates the germinal center reaction and provides a monogenic model for common variable immunodeficiency. Blood.107:3045–3052.

   [E2] Ajdukiewicz AB, Youngs GR, Bouchier IA. 1972. Nodular lymphoid hyperplasia with hypogammaglobulinaemia. Gut. 13(8):589-95.

   [E3] [Houseman M](http://www.ncbi.nlm.nih.gov/pubmed/?term=Houseman%20M%5BAuthor%5D&cauthor=true&cauthor_uid=22314025)., [Potter C](http://www.ncbi.nlm.nih.gov/pubmed/?term=Potter%20C%5BAuthor%5D&cauthor=true&cauthor_uid=22314025)., [Marshall N](http://www.ncbi.nlm.nih.gov/pubmed/?term=Marshall%20N%5BAuthor%5D&cauthor=true&cauthor_uid=22314025). et al. 2012: Baseline serum MMP-3 levels in patients with Rheumatoid Arthritis are still independently predictive of radiographic progression in a longitudinal observational cohort at 8 years follow up. [Arthritis Res Ther.](http://www.ncbi.nlm.nih.gov/pubmed/22314025);14(1):R30 [↑](#endnote-ref-1)
